# Clinical Cholecystitis in the Absence of the Gallbladder

**DOI:** 10.7759/cureus.1834

**Published:** 2017-11-10

**Authors:** Huda Naim, Syed Askari Hasan, Sameen Khalid, Aamer Abbass, Jason DSouza

**Affiliations:** 1 Internal Medicine, Dow Medical College, Karachi, Pakistan; 2 Internal Medicine Residency, Florida Hospital-Orlando

**Keywords:** gallbladder, acute cholecystitis, congenital

## Abstract

The congenital absence of the gallbladder (CAG) is a rare condition with an incidence of 13-65 cases/ 100,000 in the general population. This occurs when the gallbladder and the cystic duct fail to bud from the common bile duct during the fifth week of gestation. Most commonly, the patients with congenital absence of the gallbladder are asymptomatic. When symptomatic, they present as biliary colic, dyspepsia, jaundice or very rarely as acute cholecystitis. We present a case of a 27-year-old female who presented with acute right upper quadrant abdominal pain. Further evaluation with an ultrasound revealed a contracted gallbladder with stones. The hepatobiliary iminodiacetic acid scan was significant for non-visualization of the gallbladder, consistent with cystic duct obstruction. The laparoscopic cholecystectomy was attempted, however, the gallbladder was not visualized, and the procedure was aborted. The post-operative magnetic resonant cholangiopancreatography was consistent with the diagnosis of congenital absence of gallbladder.

## Introduction

The congenital absence of the gallbladder (CAG) is a rare entity and is estimated to occur in one in 6000 live births [1]. This anomaly was first explained by Lemery in 1701. It occurs when the gallbladder and cystic duct fail to bud from the common bile duct during the fifth week of gestation [2]. The incidence is higher in females (3:1 female to male ratio) and typically presents in the third or fourth decade of life [3]. The patients who were diagnosed with CAG were classified into three categories: asymptomatic, symptomatic and those with multiple fetal anomalies.

Most commonly, the CAG remains asymptomatic. However, there have been multiple case reports, wherein the patients have presented with right upper quadrant abdominal pain, nausea, vomiting, fatty food intolerance, dyspepsia and jaundice [4], thus highlighting the potential for the misdiagnosis as symptomatic cholelithiasis or acute cholecystitis. The diagnosis also poses a challenge because ultrasound findings are not always conclusive. Owing to this, the patients may unnecessarily undergo a surgical evaluation, if not preceded by extensive evaluation with the magnetic resonance cholangiopancreatography (MRCP) or endoscopic retrograde cholangiopancreatography (ERCP). The laparoscopy might also be needed for confirmation of the diagnosis. We present a case of a young female who presented with symptoms of biliary colic but was subsequently diagnosed with a congenital absence of the gallbladder.

## Case presentation

A 27-year-old Hispanic female presented to the hospital with complaints of the right upper quadrant (RUQ) abdominal pain for three days. The pain was constant, dull in nature, non-radiating, and worsened after consuming fatty meals. She did not have any associated constitutional symptoms. The patients' vital signs were as follows: temperature was 98.3 F, heart rate was 66 beats per minute, blood pressure was 132/87 mmHg, respiratory rate 16 breaths per minute and the oxygen saturation was 98% on room air. The physical examination was remarkable for RUQ tenderness and a positive Murphy’s sign. The laboratory data revealed a hemoglobin of 13.4 g/dL, white cell count of 6.3 x 10^9^/L, platelets of 190 x 10^9^/L, alanine transaminase of 39 IU/L, aspartate transaminase of 31 IU/L, alkaline phosphatase of 72 IU/L and a total bilirubin of 0.6 umol/L.

**Figure 1:**
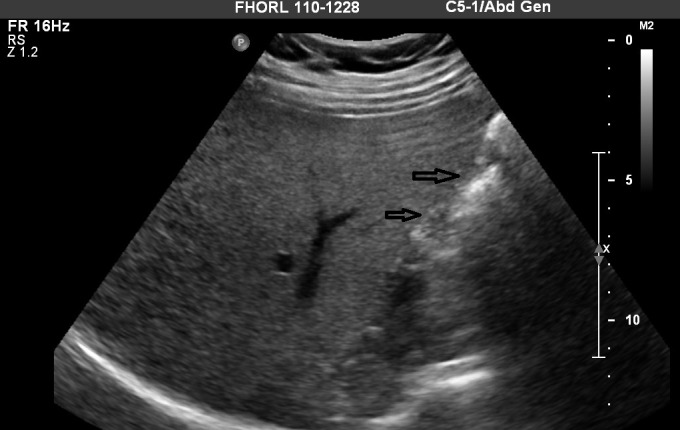
The abdominal ultrasound reported a contracted gallbladder with stones (black arrows).

**Figure 2:**
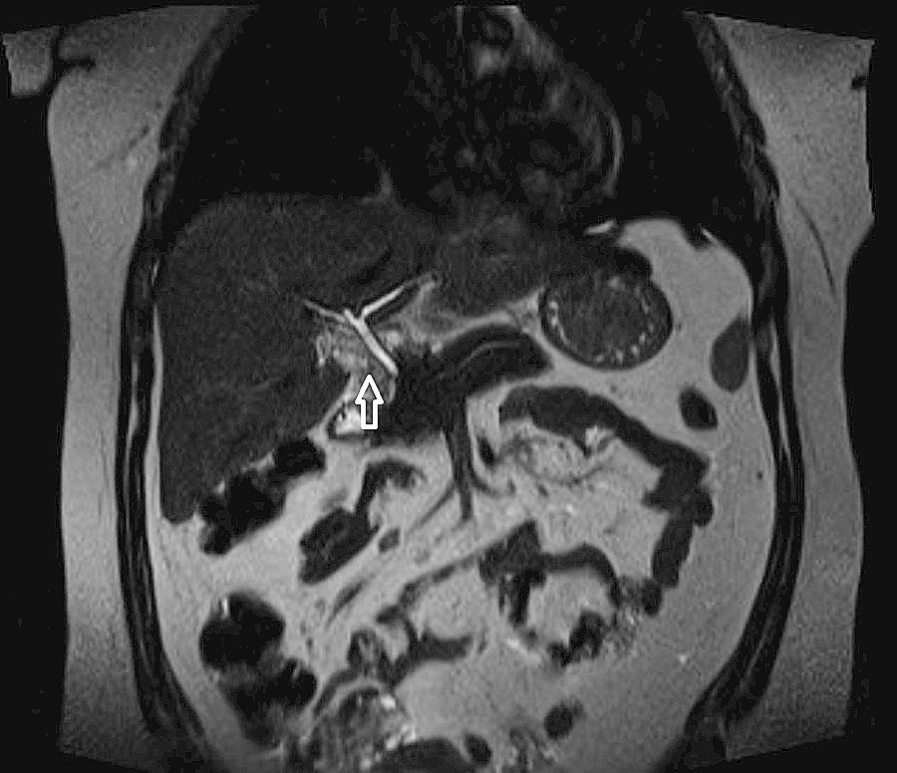
The axial image of the magnetic resonance cholangiopancreatography (MRCP) showing empty gall bladder fossa (white arrow).

The abdominal ultrasound was performed which revealed a contracted gallbladder with stones, and without any evidence for cholecystitis (Figure 1). The hepatobiliary iminodiacetic acid (HIDA) scan demonstrated non-visualization of the gallbladder, suggestive of cystic duct obstruction. With a working diagnosis of acute cholecystitis, a laparoscopic cholecystectomy was attempted, but since the gallbladder was not visualized, the procedure was aborted. Subsequently, the MRCP was performed which confirmed CAG (Figure 2). The patient’s symptoms resolved and she was discharged home on Ursodiol. There was no relapse of symptoms at the one-month follow-up visit.

## Discussion

Congenital absence of the gallbladder (CAG), which was first observed by Aristotle, is a rare condition in humans although common in many herbivorous mammals. The gallbladder develops from the caudal part of the hepatic diverticulum in the fourth week of gestation. There are two possible explanations for the agenesis. One theory is that the hepatic diverticular bud fails to develop into the gallbladder and cystic duct. While the other points towards the failure of the recanalization of cystic duct and gallbladder [1].

The CAG has a higher incidence in females as compared to males and the mean age at diagnosis is 46 years [2]. Although CAG is mostly asymptomatic, it can have variable clinical presentations. It most often presents as biliary colic, dyspepsia, or jaundice. Three groups have been identified: those with multiple fetal anomalies, asymptomatic cases, and symptomatic cases. Amongst the symptomatic, the patient's right upper quadrant pain is present in 90%, nausea and vomiting in 60%, and jaundice in 35% of the cases [1,5]. The common duct is frequently found to be dilated at exploration, usually but not necessarily in the presence of stones.

Congenital absence of the gallbladder is also associated with malformations of other systems, particularly cardiovascular, skeletal, and the abdominal wall abnormalities. This anomaly has an association with trisomy 18 and Klippel Feil syndrome and occurs because of disturbance of posterior portion of anterior diverticulum of primitive gut, involving vessels, sinus venosus, omphalocentric and umbilical veins [6].

Imaging modalities play a significant role in the diagnosis of CAG. The ultrasound is usually the initial diagnostic modality of choice in evaluating biliary pathologies owing to it being cost-effective and non-invasive. However, Fisichella, et al. and other studies [1,7] have found that the ultrasound could be misleading as the same had occurred in our case. In our patient, the ultrasound demonstrated a contracted gallbladder filled with stones, most likely mistaking the duodenal loop present in the gallbladder fossa to be the gallbladder.

The HIDA scan could also be misleading as non-visualization of the gallbladder, which serves as the the typical finding in acute cholecystitis and mucocele, often prompting a surgical approach to the management. In addition, false-positive studies have also been documented in various clinical settings, including chronic cholecystitis, cystic-duct obstruction by tumor, prolonged fasting, pancreatitis, alcoholism, parenteral hyperalimentation, and severe intercurrent illnesses [7].

It is not uncommon that most symptomatic patients with CAG undergo surgery with a presumptive diagnosis of chronic cholecystitis based on the ultrasound findings of a contracted fibrosed gallbladder. The failure to visualize the gallbladder at laparoscopy prompts surgeons to undertake a laparoscopic or open exploration of the biliary tracts thus exposing the biliary tree to the possibility of iatrogenic injury.

The MRCP is the diagnostic modality of choice as it helps in ruling out ectopic gallbladder and provides the definitive diagnosis. The widespread availability of non-invasive imaging techniques and improvement in operator skill for studies such as MRCP, ERCP and endoscopic ultrasound (EUS) have rendered these imaging modalities to be an excellent alternative to open exploration and intraoperative cholangiography [1,8]. The EUS has also been shown to be useful in detecting anomalies in the extrahepatic biliary tree [9]. These advanced imaging studies when performed prior to the surgery come at an acceptable cost compared to the morbidity and mortality associated with the surgery [4].

A probable mechanism of biliary colic in CAG is biliary dyskinesia, owing to the increased tonicity of the sphincter of Oddi in the absence of a gallbladder. This is comparable to the postcholecystectomy syndrome. The ursodeoxycholic acid has been noted to be superior to placebo in relieving the symptoms of biliary dyskinesia and may potentially have a role in CAG as well [10].

## Conclusions

The congenital absence of the gallbladder can potentially be misdiagnosed as cholelithiasis or acute cholecystitis, prematurely prompting a surgical evaluation and the possible iatrogenic insult to the biliary tree associated with the surgery. We thus recommend the use of widely available, advanced, and noninvasive imaging techniques such as MRCP prior to surgery to accurately diagnose the anatomical malformation and the associated pathology, which may then be treated accordingly. Although there are no guidelines available to treat the patients with CAG, various case reports have demonstrated resolution of symptoms with a trial of ursodeoxycholic acid.
